# Effectiveness and safety of bedaquiline-containing regimens for treatment on patients with refractory RR/MDR/XDR-tuberculosis: a retrospective cohort study in East China

**DOI:** 10.1186/s12879-022-07693-9

**Published:** 2022-08-29

**Authors:** Shao-Jun Zhang, Yan Yang, Wen-Wen Sun, Zhong-Shun Zhang, He-Ping Xiao, Yu-Ping Li, Zhe-Min Zhang, Lin Fan

**Affiliations:** 1 Department of Tuberculosis, Shanghai Pulmonary Hospital, Tongji University School of Medicine, Shanghai Clinic and Research Center of Tuberculosis, Shanghai Key Lab of Tuberculosis, Shanghai, 200433 China; 2 Department of Pharmacy, Shanghai Pulmonary Hospital, Tongji University School of Medicine, Shanghai, 200433 China; 3 Shanghai Pulmonary Hospital, Tongji University School of Medicine, Shanghai, 200433 China

**Keywords:** BDQ, Multidrug-resistant, Rifampicin-resistant, Refractory tuberculosis

## Abstract

**Objective:**

Refractory rifampicin-resistant/multidrug resistant/extensively-drug resistant tuberculosis (RR/MDR/XDR-TB) were defined as patients infected with *Mycobacterium tuberculosis* (MTB) resistant to rifampicin(RR-TB), or at least resistant to rifampicin and isoniazid (MDR-TB) or added resistant to fluoroquinolones (FQs) and one of second line injectable agents (XDR-TB), a patient for whom an effective regimen (fewer than 4 effective agents due to adverse events (AEs) or multiple drug resistances) cannot be developed. To compare the effectiveness and safety of bedaquiline (BDQ)-containing and BDQ-free regimens for treatment of patients with refractory RR/MDR/XDR-TB.

**Methods:**

Patients with refractory RR/MDR/XDR-TB receiving BDQ-containing regimens (BDQ group, n = 102) and BDQ-free regimens (non-BDQ group, n = 100) satisfied with included criteria were strictly included in this retrospective historical control study across East China. Culture conversion, treatment outcome, cavity closing rate, and AEs were compared between two groups.

**Results:**

The baseline characteristics involved all possible aspects of patients were well balanced between two groups (p > 0.05). Culture conversion rates in the BDQ group at month 3 (89.2% vs. 66.0%), month 6 (90.2% vs 72.0%), month 9 (91.2% vs. 66.0%), and month 12 (94.1% vs 65.0%) were all significantly higher than those in non-BDQ group (*p* < 0.001). Similar results were observed in the cavity closing rate at month 9 (19.6% vs 8.0%, *p* = 0.0) and month 12 (39.2% vs 15.0%, *p* < 0.001). Patients receiving BDQ-containing regimens had more treatment success than those receiving BDQ-free regimens (*p* < 0.001; cure rate, 69.6% vs. 45.0%; complete the treatment, 22.5% vs. 18.0%; treatment success, 92.2% vs. 63.0%); the use of BDQ and combined with Linezolid or Clofazimine or Cycloserine were identified as independent predictors of treatment success and no culture reversion (P < 0.05). AEs were similarly reported in 26.5% of patients in the BDQ group and 19.0% in the non-BDQ group (*p* = 0.2).

**Conclusions:**

BDQ-containing regimens resulted in better treatment outcomes and similar safety relative to BDQ-free regimens for patients with refractory pulmonary RR/MDR/XDR-TB.

**Supplementary Information:**

The online version contains supplementary material available at 10.1186/s12879-022-07693-9.

## Introduction

Drug-resistant tuberculosis (DR-TB) remains a public health burden and represents a serious threat to patients, communities and health care services [1]. The World Health Organization (WHO) estimated that there were 465,000 incident cases of rifampicin-resistant tuberculosis (RR-TB) in 2019, and 78% had multidrug-resistant tuberculosis (MDR-TB) [2]. However, the WHO reports that treatment outcomes of MDR -TB remain poor, with a treatment success rate of 57% [2].

Therefore, novel and effective anti-TB agents are urgently needed to improve the treatment outcomes of RR/MDR-TB.

BDQ is a new anti-TB agent that has been confirmed to improve treatment outcomes and culture conversion rates when added to conventional MDR-TB treatment regimens [3, 4]. Based on the above evidence, BDQ has been recently recommended by the WHO as an initial drug for use in all-oral MDR-TB treatment regimens [5]. Nevertheless, for patients with MDR-TB that is refractory to treatment due to multiple drug resistance or intolerance leading to treatment discontinuation, treatment is extremely complex [6]. In this group of patients, it is challenging to compose treatment regimens that contain at least four likely effective agents [7]. In China, BDQ was introduced into routine care for the treatment of MDR-TB in 2018 [8]. Therefore, this retrospective cohort study aimed to explore the effectiveness and safety with BDQ-containing regimens for the patients with refractory RR/MDR/XDR-TB, compared to those receiving BDQ-free regimens, the end-of-treatment outcomes and 6-month culture conversion rates of the patients receiving an all-oral bedaquiline-based regimen, injectable-containing but bedaquiline-free regimen, or injectable and bedaquiline-containing regimen were evaluated.

## Materials and methods

### Study design and patients

The retrospective cohort study across East China (Shanghai, Anhui, Zhejiang, Jiangxi and Jiangsu province) was analyzed the treatment outcomes of patients with refractory MDR/RR-TB receiving BDQ-containing regimens during August 2018- August 2020 (BDQ group), and compared to those receiving BDQ-free regimens between August 2016 and July 2018 (non-BDQ group). Ethics approval was obtained from the Institutional Review Board of Shanghai Pulmonary Hospital (number: 114JH for BDQ group and k17-138 for non- BDQ group).

Refractory RR/MDR/XDR-TB was defined as a patient infected with *Mycobacterium tuberculosis* (MTB) that was resistant to both rifampicin and isoniazid (MDR-TB) or rifampicin (RR-TB) or XDR-TB (extensively drug-resistant TB), a patient for whom an effective regimen (fewer than 4 effective agents due to AEs or multiple drug resistances) cannot be developed. RR-TB was defined as patients with MTB resistant to rifampicin, XDR-TB was defined as patients with MDR-TB having additional resistant to FQs and second-line injectable agents as previous definition of WHO guidelines. The inclusion criteria of patients were as follows: (1) patients with RR/ MDR/XDR-TB confirmed by sputum culture and drug susceptibility tests (DST or Xpert MTB/RIF); (2) patients for whom effective regimens (fewer than 4 effective agents due to intolerance, discontinuation of a second-line drug regimen due to AEs or multiple drug resistances) could not be developed; and (3) patients who consented to participate in the study and signed the consent form. Patients with severe heart, liver, lung or kidney dysfunction or failure, malignant tumors and serum HIV positive were excluded from the study whatever BDQ group or non-BDQ group; patients with BDQ allergy, Fridericia-corrected QT (QTcF) interval > 450 ms, or significant electrocardiograph abnormalities at screening were excluded from the BDQ-group.

### Treatment regimens

The treatment regimens of all patients were uniformly formulated by specialists group in Shanghai Pulmonary Hospital and managed by the Shanghai Municipal Center for Disease Control and Prevention. According to WHO guidelines, individualized treatment regimens were designed for patients based on their previous histories of anti-TB treatment and DST results. The total treatment duration was 18–20 months and follow-up after finishing the treatment was at least six months.

In the non-BDQ group, patients with RR-TB/ MDR/XDR-TB initially started treatment with an individualized regimen of at least five anti-TB drugs in the intensive phase, including Fluoroquinolones(FQs, including levofloxacin, Lfx with dose of 600 mg once a day or moxifloxacin, Mfx with dosed of 400 mg once a day), Second-line injectables (capreomycin with dose of 750 mg once a day or amikacin with dose of 400–600 mg once a day), cycloserine (Cs) with dose of 250 mg, twice or three times a day, Prothionamide (200 mg, three times a day), pyrazinamide (500 mg, three times a day) or clofazimine (Cfz) with dose of 100–150 mg once a day, Ethambtol (E) with dose of 750 mg once a day or linezolid (Lzd) with dose of 600–1200 mg once a day or para-aminosalicylic (2 g every 6 h a day), based on the treatment history of TB and DST results.

Patients in the BDQ group received BDQ-containing regimens. BDQ was administered for 24 weeks (with a loading dose of 400 mg once a day for the initial 2 weeks, followed by 200 mg three times a week for the remaining 22 weeks). Background regimens consisted of at least 4 likely effective anti-TB drugs in the intensive phase, including fluroquinolone (Lfx or Mfx), injectable agents (capreomycin or amikacin), Cs, protionamide, E, pyrazinamide, Cfz, Lzd, or para-aminosalicylic, doses of drugs were as same as those in non-BDQ group, choosing background drugs was based on the treatment history of TB and DST results of each patients.

### Drug sensitive test

All patients were positive in BACTEC MGIT 960 culture or in Xpert MTB/RIF, isolated strain were received DST, patients with DST indicated at least resistant to H and R or R resistance by Xpert MTB/RIF were included, DST included Streptomycin (Sm), Amikacin (Ak), Capreomycin (Cm), isoniazid (H), Rifamipin (R), Ethambutol (E), Oflaxacin (Ofx), and Include Linezolid DST if done and LPA DST if done.

### Treatment evaluation

Information on the patients’ demographic characteristics, background regimens, TB treatment history, laboratory test results, sputum culture conversion, end-of-treatment outcomes, radiological findings and AEs was collected.

The primary effectiveness outcomes of interest were culture conversion and end-of-treatment outcomes. Culture conversion was defined as two consecutive negative results received at least 30 days apart as previous guidelines [9]. The sputum culture conversion status was compared between the two groups at 3, 6, 9, and 12 months following treatment initiation. Culture reversion was defined as two consecutive positive sputum cultures at least 30 days apart after initial culture conversion [10]. The end-of-treatment outcomes were assigned according to WHO definitions [11], including treatment success (cure or treatment completion), treatment failure, loss to follow-up, and death. The end-of-treatment outcomes and 6-month culture conversion rates of the patients receiving an all-oral bedaquiline-based regimen, injectable-containing but bedaquiline-free regimen, or injectable and bedaquiline-containing regimen were evaluated.

Imaging evaluation (Chest CT scanning) was performed every 3 months during the treatment duration. The cavity closing rate was calculated by dividing the number of patients who had cavity closure by the number of patients found to have cavitary lesions at baseline in each group.

According to the TB treatment history, patients could be classified into new cases (patients who had never been treated for TB or had taken anti-TB medicines for less than 1 month) and previously treated cases (patients who had received anti-TB medicine more than 1 month in the past). In addition, previously treated cases were further divided into: Patients with a one-time of drug-sensitive TB treatment and failed or relapsed; Patients with a two-time of TB treatment history and failed or relapsed at the most recent treatment; Patients with a three-time TB treatment history and failed or relapsed at the most recent treatment.

AEs of interest were mainly evaluated by laboratory-monitored parameters (leucopenia, liver function, renal function, etc.) and electrocardiography (QTcF interval prolongation). Significant QTcF interval prolongation was defined as any QTcF interval value ≥ 500 ms or any increase in QTcF interval value > 60 ms from the baseline [12]. Serious adverse events (SAEs) was defined as patients occurring deaths, life-threatening status, disability or dysfunction forever or seriously, need hospitalization or extended staying in hospital, congenital malformation or abnormal.

### Statistical analysis

Quantitative variables were expressed as the means ± standard deviations (SD) or median interquartile range (IQR) values and compared using Student’s t-test. Qualitative data were expressed as numbers and percentages and evaluated using the Chi-square (χ^2^) test including the differences of the end-of-treatment outcomes and 6-month culture conversion rates among treatment groups. The median times of culture conversion and reversion were estimated using the Kaplan–Meier method and compared across different regimens using a log-rank test. Logistic regression was used to identify the independent predictors associated with end-of-treatment success and culture reversion. QTcF is QT interval corrected using the Fridericia formula. Statistical analyses were performed using SPSS version 26.0 (SPSS Institute, IL, USA), and statistical significance was set at *p* < 0.05.

## Results

### Patient characteristics

Overall, 202 eligible patients with refractory MDR/RR-TB were included in this retrospective study. Among the 202 patients, 102 were treated with BDQ-containing regimens (BDQ group), and they had a median age of 37.0 years (IQR, 28.0–52.0). The other 100 patients (median age, 43.5 years; IQR, 29.0–54.0) were treated with BDQ-free regimens (non-BDQ group). The two groups were generally well balanced for age, sex, cavitary disease, extrapulmonary TB, diabetes, TB treatment history, drug resistance type and the distribution of resistant to drugs especially second-line drugs (all *p* > 0.05). Among 8 cases of RR-TB, there were 1 case in BDQ group and 4 cases in non-BDQ group who were confirmed by Xpert MTB/RIF lacking of results of culture DST, in addition, there was 1 case in non-BDQ group who was confirmed by culture DST with resistance to Sm and R, 2 cases in BDQ group confirmed by culture DST with resistance to Sm and R, resistance to Ak, Sm, Ofx and E, respectively. The baseline characteristics of the included patients are listed in Table 1.

### Background regimens

The background drugs included in the treatment regimens of patients from the two groups are presented in Additional file 1: Fig. S1. In the BDQ group, the most frequently used background drugs included pyrazinamide (102/102, 100%), linezolid (92/102, 90.2%), clofazimine (92/102, 90.2%), cycloserine (77/102, 75.5%), para-aminosalicylic acid (72/102, 70.5%) and protionamide (60/102, 58.8%). Meanwhile, cycloserine (90/100, 90.0%), capreomycin (81/100, 81.0%), protionamide (72/100, 72.0%) and clofazimine (51/100, 51.0%) were the major components in non-BDQ group.

### Sputum culture conversion

**Fig. 1:**
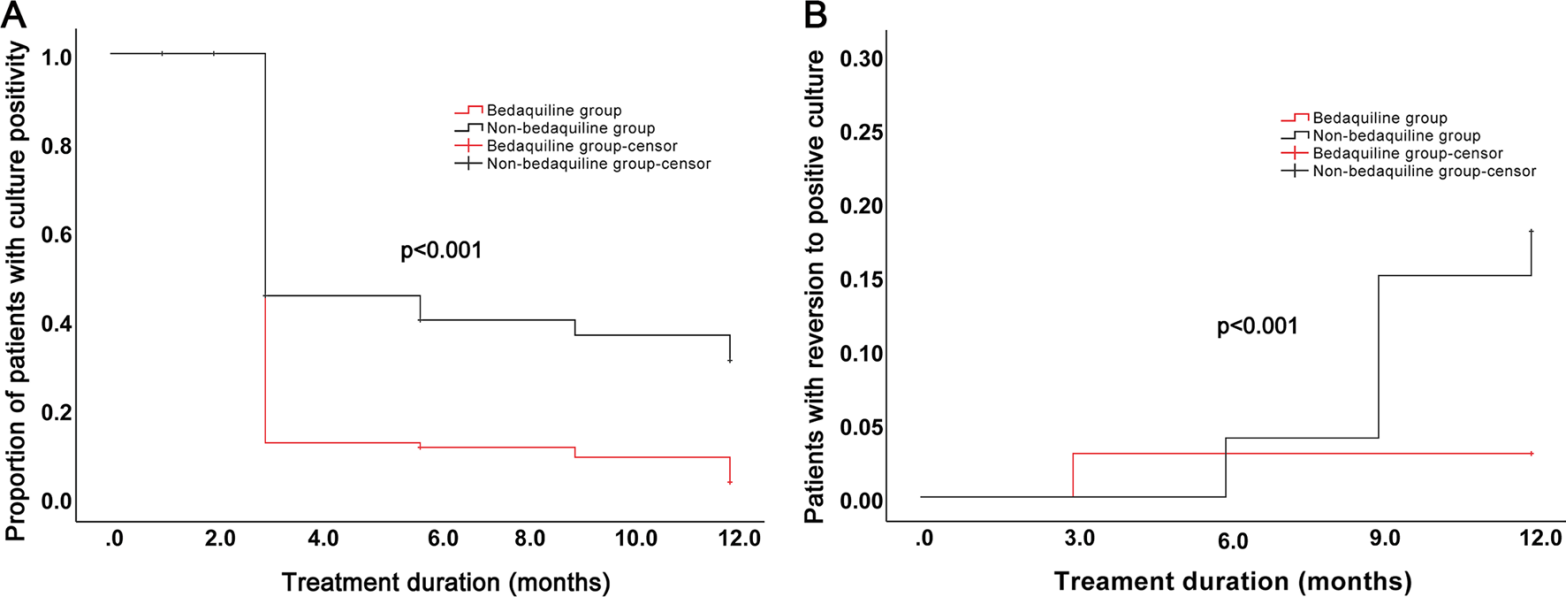
Sputum culture conversion of patients receiving BDQ-containing and BDQ-free regimens. **A** Kaplan–Meier survival curves for sputum culture conversion. **B** Kaplan–Meier survival curves for culture reversion

Among the 102 patients with culture positivity at baseline in the BDQ group, culture conversion rates of 89.2% (91/102), 90.2% (92/102), 91.2% (93/102) and 94.1% (96/102) were reported at months 3, 6, 9, and 12, respectively. Of the 100 patients with culture positivity at baseline in the non-BDQ group, 66.0% (66/100), 72.0% (72/100), 66.0% (66/100), and 65.0% (65/100) achieved culture conversion at months 3, 6, 9, and 12, respectively. Significant differences were found at all time-points between the two groups (all *p* < 0.001). In addition, the median time of culture conversion in the BDQ group was 3.0 months (IQR, 3.0–3.0), which was significantly lower than that in the non-BDQ group (5.8 months [IQR, 3.0–12.0]; *p* < 0.001; Fig. 1A). In addition, patients in the BDQ group had a lower rate of reversion to positive culture than those in the non-BDQ group (2.9% vs. 18.0%, *p* < 0.001; Fig. 1B).

Subgroup analyses of the drug resistance type or TB treatment history on the 6-month culture conversion rate of the two groups is shown in Table 2. The 6-month culture conversion rate among patients with MDR-TB in the BDQ group was significantly higher than that for patients in the non-BDQ group (87.8% vs. 73.5%, *p* < 0.05). However, significant differences were not found in the 6-month culture conversion rate of patients with RR-TB or XDR-TB between the two groups (all *p* > 0.05). In addition, patients with a ≥ three-time TB treatment history had a higher 6-month culture conversion rate in the BDQ group than in the non-BDQ group (86.4% vs. 55.2%, *p* < 0.05). The 6-month culture conversion rate among patients with new onset, one-time TB treatment history or two-time TB treatment history did not show significant differences between the two treatment groups (all *p* > 0.05).

### Treatment outcomes

Next, the end-of-treatment outcomes of patients receiving BDQ-containing regimens or BDQ-free regimens were evaluated. Among the patients in the BDQ group, 94 (92.2%) had a successful TB treatment outcome (cure, n = 71; treatment completion, n = 23), 3 (2.9%) experienced treatment failure, 4 (3.9%) were lost to follow-up and 1 (1.0%) died. Besides, treatment success (cure, n = 45; treatment completion, n = 18), treatment failure, lost to follow-up and death in the non-BDQ group were reported in 63 (63.0%), 27 (27.0%), 9 (9.0%) and 1 (1.0%) patients, respectively. Significant differences were found between the two groups (*p* < 0.001). Among the patients who were treated successfully, the median duration of the treatment was 18.0 (IQR, 18.0–24.0) and 24.0 (IQR, 24.0–24.0) months in the BDQ group and non-BDQ group, respectively.

As shown in Table 3, the proportions of patients achieving treatment success were significantly different in patients with MDR-TB (93.2% vs. 69.1%, *p* < 0.001) and XDR-TB (88.0% vs. 51.9%; *p* < 0.05) between the two groups. In addition, patients with one-time (91.9% vs. 72.0%, *p* < 0.05), two-time (90.6% vs. 71.1%, *p* < 0.001) and ≥ three-time TB treatment histories (95.5% vs. 41.4%, *p* < 0.001) in the BDQ group had higher proportions of treatment success than those in the non-BDQ group. However, after separated analysis, patients with RR/MDR-TB and XDR-TB having more than three-time history of TB treatment in BDQ group had more success rate than those patients in Non-BDQ group, however, patients with RR/MDR-TB and XDR-TB having more than three-time history of TB treatment in BDQ group had no higher sputum conversion rate than in patients with Non-BDQ group (p > 0.05) although it seemed like those patients in BDQ-group had slight higher conversion rate than those in Non-BDQ group.

### Cavity closing rate

The cavity closing rates of patients in the BDQ group at month 9 (19.6% vs. 8.0%, *p* = 0.02) and month 12 (39.2% vs. 15.0%, *p* < 0.001) were significantly higher than those in the non-BDQ group. However, no significant differences were found in the cavity closure rate at month 3 (2.9% vs. 2.0%, *p* = 1.00) or month 6 (11.8% vs. 4.0%, *p* = 0.07) between the two groups.

### Independent predictor of treatment success and culture reversion

Univariate and multivariate analyses showed that the use of BDQ was an independent predictor of both treatment success (OR = 7.4, 95% CI: 2.9–18.5, *p* < 0.001) and culture reversion (OR = 0.1, 95% CI: 0.0–0.5, *p* < 0.001) after adjusting for age, sex, cavitary disease, diabetes, TB treatment history, and drug resistance type. Besides use of BDQ, use of BDQ combined with Cfz, Lzd and Cs, respectively were independent predictor of treatment success in univariate and multivariate analyses (OR was 13.0, 95%CI (4.4 -38.1), *p* < 0.001, OR was 13.7, 95%CI (1.8–103.8), p = 0.01 and OR was 13.1, 95%CI (3.7- 45.7), *p* < 0.001, respectively. Similarly, use of BDQ combined with Cfz, Lzd and Cs, respectively were independent predictor of less culture reversion, OR were < 0.001, 0.001 and 0.001. However, use of BDQ combined with FQs in multivariate analysis was not independent predicator of treatment success and culture reversion, P was 0.27 and 1.0. To further compare the combination of BDQ and two or three core drugs between two groups, we found that BDQ + injectable agents had better outcome (success rate or culture conversion)than patients with absence of BDQ combinations, BDQ + Lzd + FQs or BDQ + Lzd + FQs + Cfz had better success rate than those in controlled group while the other combinations had no obvious impact on success rate; for culture reversion, BDQ + Lzd + FQs or BDQ + Lzd + FQs + Cfz and other combinations had no obvious impact in culture reversion between two groups no matter on Univariate analysis or multivariate analysis (p > 0.05). There were too small cases of patients using combination of BDQ with more than other three or four core drugs, thereby part of data shown might be not efficient. The detailed data were shown in (Tables 4, 5).

### AEs

In the BDQ group, 27 patients (26.5%) reported a total of 37 AEs during the total treatment duration. The most commonly reported AEs were nephrotoxicity (n = 12, 11.8%), hepatotoxicity (n = 9, 8.8%), peripheral neuropathy (n = 3, 3.0%), leukopenia (n = 2, 2.0%), hypokalemia (n = 2, 2.0%), ototoxicity (n = 2, 2.0%), and others (n = 4, 3.9%). 3 caes in BDQ group and 2 cases in Non-BDQ group had slightly QTcF interval prolongation (< 450 ms) was observed (Table 6).

In the non-BDQ group, a total of 26 AEs were observed in 19 patients (19.0%). The most frequently observed AEs were ototoxicity (n = 8, 8.0%), nephrotoxicity (n = 3, 3.0%), hepatotoxicity (n = 2, 2.0%), leukopenia (n = 2, 2.0%), hypokalemia (n = 1, 1.0%), peripheral neuropathy (n = 1, 1.0%), and others (n = 7, 7.0%).

Significant differences were not observed in the occurrence of AEs reported in these two groups (*p* = 0.2). In addition, no SAEs were observed in any patients from the two groups.

### Differences in treatment outcomes among different regimens

**Fig. 2:**
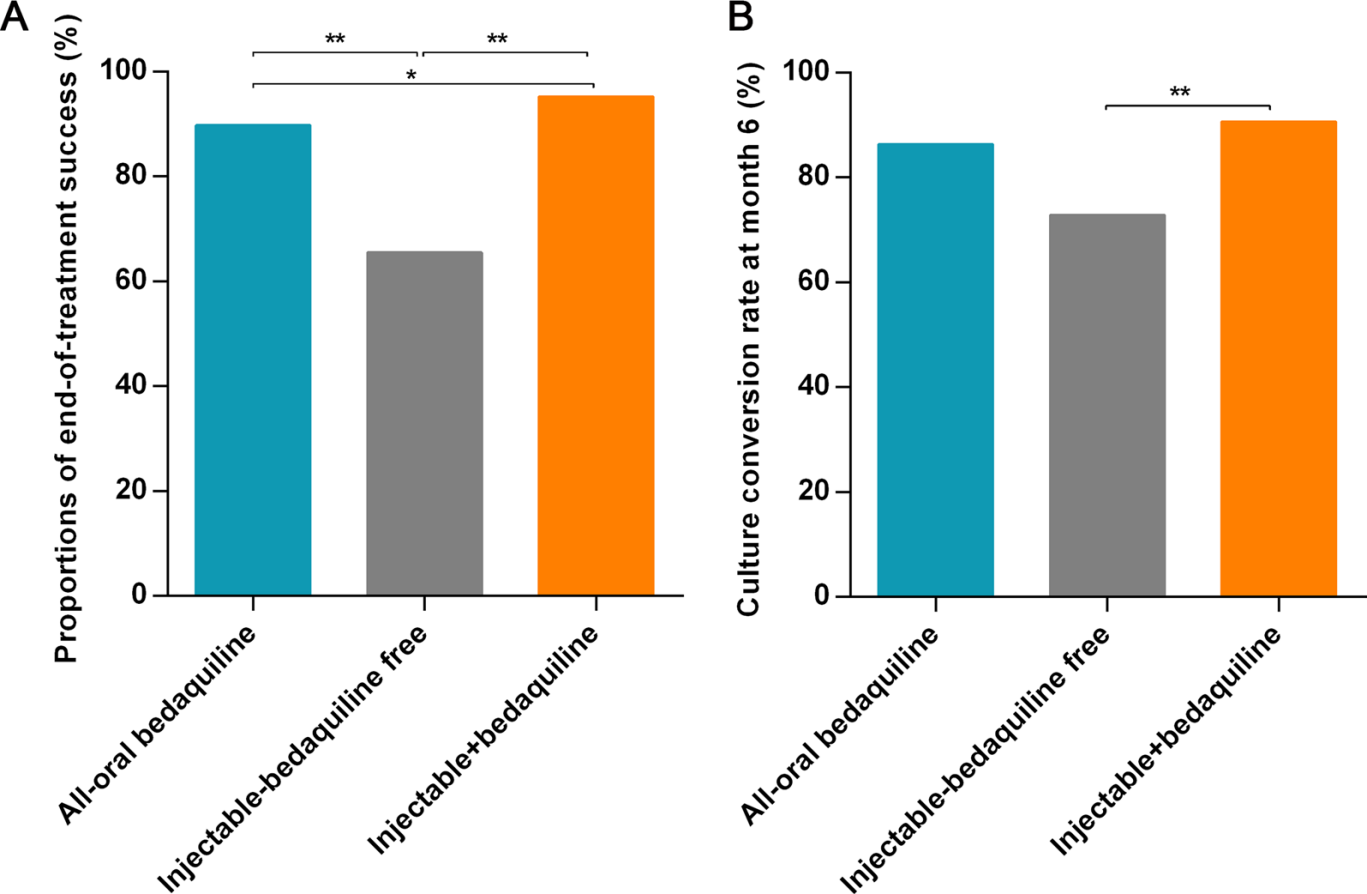
Treatment outcomes of patients receiving different regimens. **A** Proportions of end-of-treatment success of patients receiving different regimens. **B** Culture conversion rate at month 6 of patients receiving different regimens. *p < 0.05; **p < 0.01

Subsequently, the end-of-treatment outcomes and 6-month culture conversion rates of the patients receiving an all-oral BDQ-based regimen, injectable-containing but BDQ-free regimen, or injectable and BDQ-containing regimen were evaluated. As shown in Fig. 2A, B, significant differences were observed between the three treatment groups in the proportions of end-of-treatment success (89.8% vs. 65.5% vs. 95.3%, *p* < 0.001) and the 6-month culture conversion rate (86.4% vs. 72.9% vs. 90.7%, *p* = 0.02).

## Discussion

The End TB strategy of the WHO aims to end the global TB epidemic by 2035 [13]. However, compared to drug-susceptible TB, current treatment outcomes for DR-TB are relatively poor, especially for patients with refractory RR/MDR/XDR-TB [14]. Moreover, treatment options for refractory RR/MDR/XDR-TB are extremely limited. Patients often experience failed DR-TB treatment and present complicated medical histories of second-line drugs or intolerance to several effective drugs; therefore, treatment regimens often rely on a set of drugs with a high pill burden, poorly established efficacy and severe AE profiles [2]. In some settings, poor efficacy and tolerability might lead to the discontinuation of treatment, which increases patient mortality and transmission risk of highly resistant strains [15]. Therefore, effective and safe treatment regimens for refractory RR/MDR/XDR-TB must be developed for the realization of the WHO’s End TB strategy.

BDQ is an ATP synthase inhibitor and a novel agent recommended by the WHO for the treatment of MDR-TB [16]. Adding BDQ to standard treatment regimens for MDR-TB has been shown to improve the long-term survival of patients, reduce the time for sputum culture conversion, and increase the proportion of patients with consecutive negative culture results [17–19]. Given that patients with refractory RR/MDR/XDR-TB have few treatment options, BDQ might represent a useful choice for this population. However, evidence about the effectiveness and safety of BDQ-containing regimens in this group of patients is lacking. Therefore, we conducted a retrospective cohort study and compared the treatment outcomes of BDQ-containing and BDQ-free regimens for the treatment of patients with refractory RR/MDR/XDR-TB in East China.

The present study showed that patients receiving BDQ-containing regimens in the present study had higher culture conversion rates at month 6 (90.2% vs. 72.0%) and treatment success rates (92.2% vs. 63.0%) than those receiving BDQ-free regimens. Similarly, in the previous studies the culture conversion rate at month 6 in patients treated with BDQ-containing regimens varied from 64 to 100% and the treatment success rate ranged between 52 and 85% [19–21]. Both outcomes were higher in patients treated with BDQ than in those without BDQ. Moreover, the use of BDQ was identified as an independent predictor of treatment success (OR = 7.356, 95% CI: 2.920–18.530, *p* < 0.001), thus reflecting the superior antibacterial and bactericidal activity of BDQ against tubercle bacilli [22]. Interestingly, the treatment success rate observed in our patients treated with BDQ was slightly higher than that in previous reports, which may be related to the background drugs included in the BDQ-containing regimens. Nearly all participants in the BDQ group received pyrazinamide (100%), linezolid (90.2%), and clofazimine (90.2%). Evidence has shown that linezolid has a positive effect on the clinical outcomes of patients with DR-TB [23, 24]. The addition of pyrazinamide to BDQ-clofazimine combination regimens resulted in even more rapid sterilizing activity [25, 26]. In the present study, our data also showed that BDQ combined used with Lzd, or Cfz, or Cs were the independent predicative factors of treatment success and culture reversion. The reason why BDQ combined with FQs in multivariate analysis was not independent predicative factor of treatment outcome was possible due to less number (23) of cases were combined BDQ with FQs, more samples should be analysed further in the future study. Taken together, the inclusion of BDQ in the treatment regimens for patients with refractory RR/MDR/XDR-TB was associated with good outcomes.

To the best of our knowledge, this study showed that patients with more than one-time of drug sensitive TB treatment history, MDR-TB or XDR-TB were more likely to benefit from BDQ-containing regimens and showed a higher treatment success rate than those receiving BDQ-free regimens. Patients with previous TB treatment particularly with second line TB drugs are likely to benefit from BDQ because it is likely to be effective as BDQ was not used previously. The frequency of drug resistance for anti-TB drugs of previously treated TB was found to be five times higher than that of new-onset patients [27]. An increase in resistant to anti-TB drugs against *Mycobacterium tuberculosis* poses a major threat to TB control [28]. In addition, compared with RR-TB, MDR-TB and XDR-TB were more difficult to manage and showed broad-spectrum resistance, poor outcomes and high mortality [29]. In the present study, we found the success rates of BDQ-containing group in one time of ATT (Anti-TB treatment), two-time and more than three-time of ATT were higher than those of patients with non-BDQ group while there was no difference between groups in new case, implying the possibility that more complicated history of ATT, the more drug-resistant extent exists which had more benefit from BDQ. Overall, above findings further confirmed that BDQ-containing other drugs such as Lzd, Cfz, injectable agents or Cs regimens showed superior efficacy in treatment patients with refractory RR/MDR/XDR-TB. The results also showed that BDQ combined with FQs had slight better outcome and decreased culture reversion on univariate analysis (P values were 0.03) while had no significance differences on multivariate analysis. We know that FQs is the one of the most important drugs on the treatment for MDR-TB, the reason of its lack of significant role for Refractory MDR/XDR-TB was due to about over half of patients resistant to FQs in the present study (data shown in Table 1). Therefore, new drugs had the absolute advantage on XDR-TB or pre-XDR-TB patients while FQs had not. For the analysis of BDQ combination with two or three core drugs, the results indicated that BDQ combination with Lzd, FQs and Cs or Lzd can increase the success rate while combination with more than three core drugs had not been found to have significant impact on success rate and culture conversion, the reason was possibly due to limited cases enrolled into each combination group.

Another important finding from this study was that the end-of-treatment success proportion and 6-month culture conversion rate varied with the treatment regimen. Overall, there was a high treatment success rate and culture conversion rate for patients who received BDQ, regardless of whether an injectable agent was administered. Prior to August 2018, RR/MDR-TB treatment guidelines recommended parenteral administration of capreomycin or aminoglycoside (kanamycin or amikacin) as part of the treatment [30]. However, due to the significant toxicity and patient intolerance that led to treatment interruption [31], the WHO deprioritized the injectable-containing regimens in 2019 and recommended the use of all-oral regimens [30]. Therefore, although the treatment success rate and culture conversion rate in the present study were slightly higher in patients treated with BDQ and injectable agents than in those who did not receive injectable agents, an all-oral BDQ-based regimen was recommended for the treatment of patients with refractory RR/MDR/XDR-TB.

Of special concern was that the use of BDQ was associated with QTcF interval prolongation [32]. In our cohort, only 3 cases experienced slightly QTcF interval prolongation but no patient stopped BDQ administration, suggesting that BDQ, even in combination with clofazimine, might be safe. Other studies have reported a similarly low rate of cardiotoxicity [33, 34]. This finding could therefore alleviate some concerns about the risk of cardiotoxicity associated with the use of BDQ. The AE occurrence rates were similar between BDQ group and Non-BDQ group in term of Nephrotoxicity, Hepatotoxicity, Peripheral neuropathy, Leukopenia, Hypokalemia, Ototoxicity and others, demonstrating that regimen containing BDQ was safe.

Several limitations were observed in this study. First, this retrospective cohort study included patients receiving BDQ-containing regimens from August 2018 to August 2020 and patients receiving BDQ-free regimens from August 2016 to July 2018. Although the baseline characteristics of patients were well balanced between the two groups, the implementation conditions (monitoring, quality of care, staffing) changed over time, which might cause bias in the analyses. Second, AEs were based on self-reports and clinician notes from handwritten medical records, which is a limitation inherent in all retrospective studies. The overall low rate of AEs observed in our study could be a consequence of underreporting. However, despite the combined use of other drugs that also cause QTcF interval prolongation, the current data showed the absence of critical cardiac AEs in patients receiving BDQ, thus demonstrating the safety of BDQ under procedural conditions for treating patients with refractory RR/MDR/XDR-TB. Therefore, it is necessary to conduct prospective studies to comprehensively and accurately evaluate the effectiveness and safety of BDQ-containing regimens for the treatment of refractory RR/MDR/XDR-TB.

## Conclusions

BDQ-containing regimens resulted in better treatment outcomes and comparable safety compared to BDQ-free regimens for patients with refractory pulmonary RR/MDR/XDR-TB. The use of BDQ and combined with Lzd, Cfz or Cs were identified as independent predictors of favorable treatment outcomes. These findings provide significant positive insights in support of the recent WHO recommendations on the use of BDQ combined with other drugs.

**Table 1.** Baselines characteristics of all patients

Characteristics	Bedaquiline group (n = 102)	Non-bedaquiline group (n = 100)	*p* value
Age (years, median/IQR)	37.0 (28.0–52.0)	43.5 (29.0–54.0)	0.08
Gender (n, %), male/female	78 (76.5)/24 (23.5)	71 (71.0)/29 (29.0)	0.77
Cavitary disease at baseline (n, %)	71 (73.2)	73 (73.0)	0.65
Concomitant extra-pulmonary TB (n, %)	20 (19.6)	15 (15.0)	0.39
Concomitant diabetes (n, %)	16 (15.7)	13 (13.0)	0.59
History of TB treatment (n, %)			0.85
New cases	9 (8.8)	8 (8.1)	
Previously treated cases	93 (91.2)	91 (91.9)	
Type of drug resistance (n, %)			0.67
RR	3 (2.9)	5 (5.0)	
MDR	74 (72.5)	68 (68.0)	
XDR	25 (24.5)	27 (27.0)	
Drug resistant rate (n, %)/n, % in XDR			
Injectable agents	83 (81.4)/25 (100.0)	77 (77.0)/27 (100.0)	0.44/–
FQs	61 (59.8)/25 (100.0)	55 (55.0)/27 (100.0)	0.49/–
EMB	57 (55.9)/16 (64.0)	65 (65.0)/24 (88.9)	0.19/0.03*
INH	97 (95.1)/23 (92.0)	98 (98.0)/26 (96.3)	0.20/0.95

*IQR* interquartile range, *M* male, *F* female, *n* number, *TB* tuberculosis, *RR* rifampicin-resistant, *MDR* multidrug-resistant, *XDR* extensively drug-resistant, *FQs* fluoroquinolones, *EMB* ethambutol, *INH* isoniazid. Injectable agents: including Amikacin (Ak), Capremycin (Cm) and Streptomycin (Sm)

* the differences had statistical significance (*p* < 0.05)

**Table 2:** The effect of the type of drug resistance or history of TB treatment on the culture conversion rate at month 6 of the two groups

Indicators	BDQ group (n = 102)		Non-BDQ group (n = 100)		*p* value
	Conversion	Failed to conversion	Conversion	Failed to conversion	
Type of drug resistance (n, %)					
RR	3 (100.0)	0 (0.0)	4 (80.0)	1 (20.0)	1.00
MDR	65 (87.8)	9 (12.2)	50 (73.5)	18 (26.5)	0.03*
XDR	22 (88.0)	3 (12.0)	18 (66.7)	9 (33.3)	0.07
History of TB treatment (n, %)					
New cases	10 (90.9)	1 (9.1)	6 (75.0)	2 (25.0)	0.55
One-time history of TB treatment	34 (91.9)	3 (8.1)	19 (76.0)	6 (24.0)	0.17
Two-time history of TB treatment	27 (84.4)	5 (15.6)	31 (81.6)	7 (18.4)	0.76
≥ Three-time history of TB treatment	19 (86.4)	3 (13.6)	16 (55.2)	13 (44.8)	0.02*
MDR/RR-TB	(n = 77)		(n = 73)		
New cases	7(100.0)	0(0.0)	5 (71.4)	2 (28.6)	0.46
One-time history of TB treatment	30 (90.9)	3 (9.1)	15 (71.4)	6 (28.6)	0.06
Two-time history of TB treatment	19 (86.4)	3 (13.6)	23 (88.5)	3 (11.5)	0.83
≥ Three-time history of TB treatment	13 (86.7)	2 (13.3)	11 (57.9)	8 (42.1)	0.13
XDR-TB	(n = 25)		(n = 27)		
New cases	3 (75.0)	1 (25.0)	1 (100.0)	0 (0.0)	1.00
One-time history of TB treatment	4 (100.0)	0 (0.0)	4 (100.0)	0 (0.0)	–
Two-time history of TB treatment	8 (80.0)	2 (20.0)	8 (66.7)	4 (33.3)	0.65
≥ Three-time history of TB treatment	6 (85.7)	1 (14.3)	5 (50.0)	5 (50.0)	0.30

*TB* tuberculosis, *n* number, *RR* rifampicin-resistant, *MDR* multidrug-resistant, *XDR* extensively drug-resistant, *BDQ* bedaquiline

* the differences had statistical significance (*p* < 0.05)

**Table 3:** The effect of the type of drug resistance or history of TB treatment on the proportion of treatment success of the two groups

Indicators	BDQ group (n = 102)		Non-BDQ group (n = 100)		*p* value
	Success	Others	Success	Others	
Type of drug resistance (n, %)					
RR	3 (100.0)	0 (0.0)	2 (40.0)	3 (60.0)	0.20
MDR	69 (93.2)	5 (6.8)	47 (69.1)	21 (30.9)	< 0.001^*^
XDR	22 (88.0)	3 (12.0)	14 (51.9)	13 (48.1)	0.005^*^
History of TB treatment (n, %)					
New cases	10 (90.9)	1 (9.1)	6 (75.0)	2 (25.0)	0.55
One-time history of TB treatment	34 (91.9)	3 (8.1)	18 (72.0)	7 (28.0)	0.04^*^
Two-time history of TB treatment	29 (90.6)	3 (9.4)	27 (71.1)	11 (28.9)	0.04^*^
≥ Three-time history of TB treatment	21 (95.5)	1 (4.5)	12 (41.4)	17 (58.6)	< 0.001^*^
MDR/RR-TB	(n = 77)		(n = 73)		
New cases	7 (100.0)	0 (0.0)	5 (71.4)	2 (28.6)	0.46
One-time history of TB treatment	30 (90.9)	3 (9.1)	16 (76.2)	5 (23.8)	0.14
Two-time history of TB treatment	21 (95.5)	1 (4.5)	20 (76.9)	6 (23.1)	0.07
≥ Three-time history of TB treatment	14 (93.3)	1 (6.7)	8 (42.1)	11 (57.9)	0.003*
XDR-TB	(n = 25)		(n = 27)		
New cases	3 (75.0)	1 (25.0)	1 (100.0)	0 (0.0)	1.00
One-time history of TB treatment	4 (100.0)	0 (0.0)	2 (50.0)	2 (50.0)	0.432
Two-time history of TB treatment	8 (80.0)	2 (20.0)	7 (77.8)	5 (22.2)	1.00
≥ Three-time history of TB treatment	7 (100.0)	0 (0.0)	4 (40.0)	6 (60.0)	0.04*

*TB* tuberculosis, *n* number, *RR* rifampicin-resistant, *MDR* multidrug-resistant, *XDR* extensively drug-resistant, *BDQ* bedaquiline

* the differences had statistical significance (*p* < 0.05)

**Table 4:** Univariate and multivariate analysis of independent predictors for treatment success

Independent factors		Treatment success	Univariate analysis		Multivariate analysis	
			OR (95%CI)	*p *value	OR (95%CI)	*p *value
Age						
< 35 years	72 (85.7)		–	–	–	–
35–60 years	73 (73.0)		0.45 (0.2–1.0)	0.04	2.7 (0.6–11.4)	0.2
≥ 60 years	14 (77.8)		0.6 (0.16–2.1)	0.6	1.0 (0.3–3.6)	1.0
Type of drug resistance					–	–
RR	5 (62.5)		–	–	–	–
MDR	116 (76.3)		0.83 (0.2–3.8)	0.8	–	–
XDR	36 (58.1)		2.7 (0.6–11.9)	0.2	–	–
Gender (male/female)	114 (77.0)/42 (79.2)		0.9 (0.4–1.9)	0.8	–	–
History of TB treatment (new/previously treated)		16 (84.2)/141 (77.0)	1.6 (0.4–5.7)	0.5	–	–
Cavitary disease at baseline (yes/no)		114 (81.4)/39 (72.2)	0.6 (0.3–1.2)	0.2	–	–
Concomitant diabetes (yes/no)		21 (72.4)/136 (78.6)	1.4 (0.6–3.4)	0.5	–	–
Treatment regimen (bedaquiline/non-bedaquiline)		94 (92.2)/63 (63.0)	6.9 (3.0–15.8)	< 0.001*	7.4 (2.9–18.5)	< 0.001*
Regimen containing (BDQ+ Cfz)/Cfz		87 (94.6)/29(58.0)	12.6 (4.4– 36.4)	<0.001*	13.0 (4.4 –38.1)	< 0.001*
Regimen containing (BDQ+ Lzd)/Lzd		85 (92.4)/13 (56.5)	9.3(3.0 –28.9)	<0.001*	13.7 (1.8–103.8)	0.01*
Regimen containing (BDQ+ FQs)/FQs		19 (82.6)/45(57.7)	3.5 (1.1–11.2 )	0.03*	2.5 (0.5–12.6 )	0.27
Regimen containing (BDQ+Cs)/Cs		71(92.2)/58 (63.7)	6.7 (2.6–17.2)	< 0.001*	13.1 (3.7–45.7)	< 0.001*
BDQ+injectable agents/injectable agents		52 (96.3/60) (65.9)	13.4 (3.1–58.9)	< 0.001*	12.1 (2.7–53.7)	0.001*
BDQ + Lzd+ FQs/Lzd+FQs		14 (82.4)/9 (50.0)	4.7 (0.1–22.0)	0.08	15.2 (1.42–163.3)	0.03*
BDQ+ Lzd+ FQs+ Cfz/Cfz+ Lzd+ FQs		15 (88.2)/3 (33.3)	15.0 (2.0–113.6)	0.01*	38.9 (1.9–817.1)	0.02*

*TB* tuberculosis, *RR* rifampicin-resistant, *MDR* multidrug-resistant, *XDR* extensively drug-resistant, *95%CI* 95% confidence interval, *OR* odds ratio

* the differences had statistical significance (*p* < 0.05)

**Table 5:** Univariate and multivariate analysis of independent predictors for reversion to positive culture

Independent factors		Reversion to positive culture	Univariate analysis		Multivariate analysis	
			OR (95%CI)	*p *value	OR (95%CI)	*p *value
Age					–	–
< 35 years	5 (6.0)		–	–	–	–
35–60 years	15 (15.0)		2.8 (1.0–8.0)	0.05	–	–
≥ 60 years	1 (5.6)		1.0 (0.1–8.5)	0.94	–	–
Type of drug resistance					–	–
RR	1 (12.5)		–	–	–	–
MDR	11 (7.7)		0.6 (0.1–5.2)	0.63	–	–
XDR	9 (17.3)		1.5 (0.2–13.4)	0.73	–	–
History of TB treatment (new/previously treated)		2 (10.5)/19 (10.4)	1.0 (0.2–4.7)	0.99	–	–
Cavitary disease at baseline (yes/no)		18 (12.9)/3 (5.6)	0.4 (0.1–1.4)	0.14		
Concomitant diabetes (yes/no)		3 (10.3)/18 (10.4)	1.0 (0.3–3.7)	0.99		
Gender (male/female)	20 (13.5)/1 (1.9)		8.1 (1.1–62.1)	0.02	1.2 (0.4–4.1)	0.71
Treatment regimen (bedaquiline/non-bedaquiline)		3 (2.9)/18 (18.0)	0.1 (0.0–0.5)	< 0.001	0.1 (0.0–0.5)	0.002*
Regimen containing (BDQ+ Cfz) /Cfz		5 (5.4)/18 (36.0)	0.1(0.0–0.3)	< 0.001	0.1 (0. 0–0.4)	< 0.001*
Regimen containing (BDQ+ Lzd)/Lzd		5 (5.4)/7(30.4)	0.1 (0.0–0.5)	< 0.001	0.1(0.0–0.5)	0.001*
Regimen containing (BDQ+ FQs)/FQs		1(4.3)/19(24.4)	0.1 (0.0–1.1)	0.03	0.00 (–)	1.0
Regimen containing (BDQ+Cs)/Cs		5 (6.5)/24 (26.4)	0.2 (0.1–0.5)	0.00	0.2(0.0–0.5)	0.001*
BDQ+injectable agents/injectable agents		4(7.4)/21(23.1)	0.3 (0.1–0.8)	0.016	0.1 (0.0–0.5)	0.006*

*TB* tuberculosis, *RR* rifampicin-resistant, *MDR* multidrug-resistant, *XDR* extensively drug-resistant, *95%CI* 95% confidence interval, *OR* odds ratio

* the differences had statistical significance (*p* < 0.05)

**Table 6:** Summary of adverse events in the two groups

Groups	Nephrotoxicity (n, %)	Hepatotoxicity (n, %)	Peripheral neuropathy (n, %)	QTc prolongation (n,%)	Leukopenia (n, %)	hypokalemia (n, %)	Ototoxicity (n, %)	Others (n, %)	*p* value
Bdq group (n=102)	12 (11.8)	9 (8.8)	3 (3.0)	3(3.0)	2 (2.0)	2 (2.0)	2 (2.0)	4 (3.9)	0.21
Non-Bdq group (n=100)	3 (3.0)	2 (2.0)	1 (1.0)	2(2.0)	2 (2.0)	1 (1.0)	8 (8.0)	7 (7.0)	

## Supplementary Information

**Additional file 1: Fig. S1.** Background drugs included in the treatment regimens of patients from the two groups.

## Data Availability

All data regarding the included participants and laboratory data during the study are available from the corresponding author by email request.
