# Supplementary figures and images for: Effectiveness and safety of bedaquiline-containing regimens for treatment on patients with refractory RR/MDR/XDR-tuberculosis: a retrospective cohort study in East China
Source: BMC Infect Dis. 2022 Aug 29;22:715. doi: 10.1186/s12879-022-07693-9 (PMC9422092; doi:10.1186/s12879-022-07693-9)

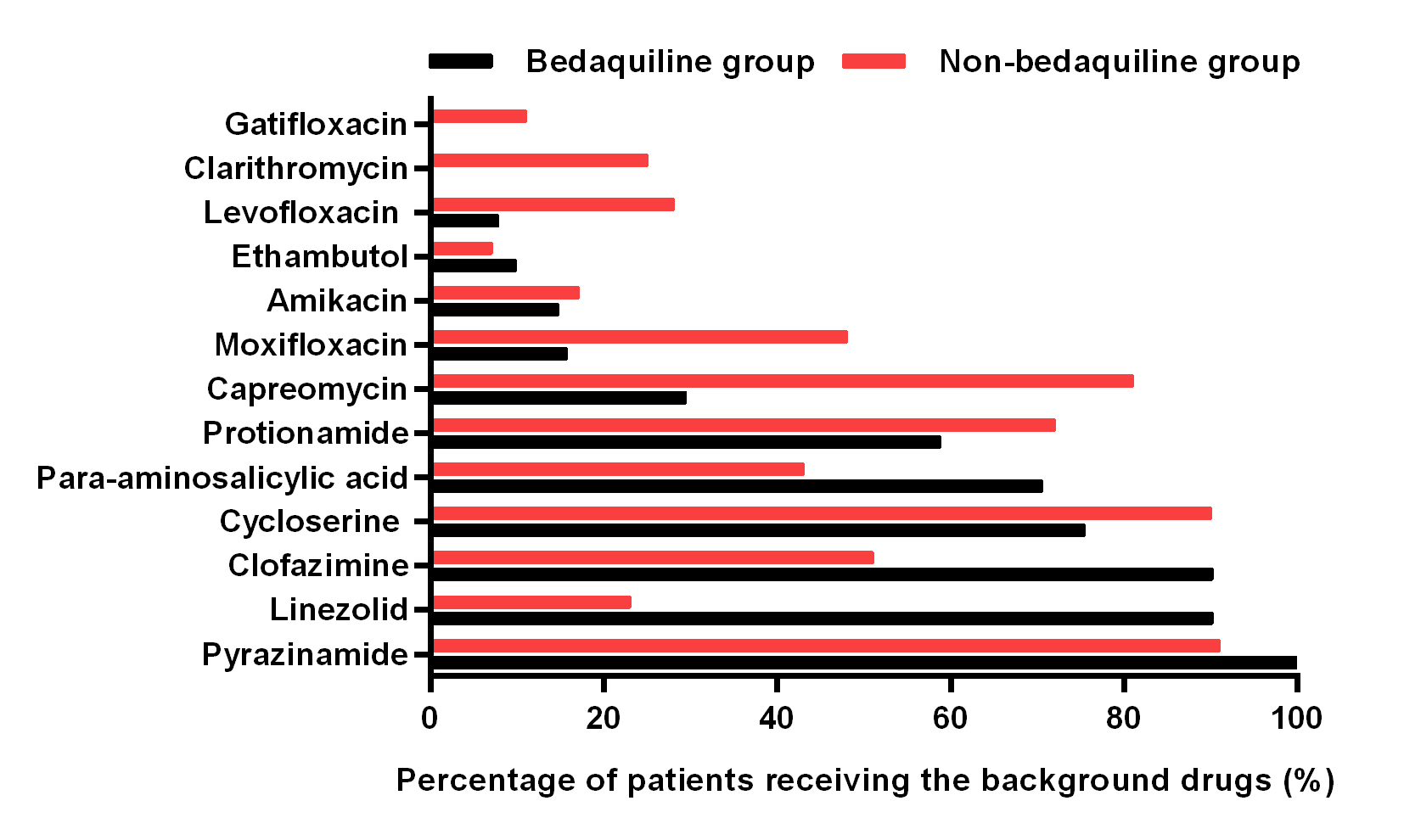

Supplement: Supplementary file 1 — Additional file 1: Fig. S1. Background drugs included in the treatment regimens of patients from the two groups. [file 12879_2022_7693_MOESM1_ESM.jpg]
